# Eosinopenia as a prognostic factor of mortality for COVID-19 in end-stage kidney disease patients

**DOI:** 10.22088/cjim.15.2.273

**Published:** 2024

**Authors:** I Gede Yasa Asmara, I Gusti Ngurah Ommy Agustriadi, I Made Sujaya, Salim Said Thalib, Rina Lestari, Suryani Padua Fatrullah, Komang Sri Rahayu Widiasari, Indana Eva Ajmala

**Affiliations:** 1 Department of Internal Medicine, Faculty of Medicine, University of Mataram - West Nusa Tenggara General Hospital, Mataram, Indonesia; 2 Department of Pulmonology, Faculty of Medicine, University of Mataram - West Nusa Tenggara General Hospital, Mataram, Indonesia

**Keywords:** COVID-19, Eosinophil, ESKD, Hemodialysis, Mortality

## Abstract

**Background:**

The unique role of eosinophil in coronavirus disease 2019 (COVID-19) patients has been shown in several studies, but its role in end-stage kidney disease (ESKD) patients who contracted COVID-19 is less reported. This study investigated eosinopenia's predictive value as a mortality marker in ESKD patients with COVID-19.

**Methods:**

It is a retrospective study of ESKD patients who contracted COVID-19 between May 2020 and October 2021 in West Nusa Tenggara General Hospital, Indonesia. Comparative analysis was carried out between the death dan survival group. Logistic regression analysis was done to investigate the role of eosinopenia on the outcome after controlling other significant variables.

**Results:**

The analyses included one hundred fifteen confirmed COVID-19 in ESKD patients. The average age was 50, 53% of patients were males, 41% were newly diagnosed with ESKD, and the mortality rate was 25.2%. This study's prevalence of eosinopenia, high neutrophil-to-lymphocyte ratio (NLR), and high C-reactive protein (CRP) in the nonsurvivors was 51.4%, 39.3%, and 30.8%, respectively. Diastolic blood pressure <90 mmHg (P=0.004), respiratory rate >22 x/minutes (P=0.011), oxygen saturation <93% (P=0.008), NLR >6 (p<0.001), eosinophil count <0.01 x10^3^/uL (p<0.001), CRP >20 mg/L (P=0.047), and isolation hemodialysis (HD) therapy (p<0.001) were independently associated with mortality of COVID-19 in ESKD patients. However, on multivariate logistic regression analysis, eosinopenia (P=0.019) and HD (P=0.001) were risk factors that remained significant prognostic mortality factors.

**Conclusion:**

Eosinopenia was common in ESKD patients with COVID-19, particularly in the death group. Eosinopenia at admission and HD during hospitalization were risk factors for COVID-19 mortality in ESKD patients.

Coronavirus disease 2019 (COVID-19) is a systemic viral infection that can attack many organs, including the kidneys. Patients with end-stage kidney disease (ESKD) are more easily infected, have more severe conditions, and have a higher mortality rate than patients without chronic kidney disease (CKD) (1, 2). The incidence of COVID-19 in patients on dialysis is higher than in patients who have not been on dialysis (1). The incidence of COVID-19 in hemodialysis (HD) patients was 7.7%, and the mortality rate was 22.4% (3). Chronic kidney disease patients with COVID-19 have a higher mortality rate than CKD patients without COVID-19 (1). Several conditions and inflammatory markers have been associated with the severity and mortality of COVID-19 in ESKD patients. Predictors of death from COVID-19 in ESKD patients include old age, multimorbidity, decreased eosinophils, increased C-reactive protein, raised D-dimer, and increased lactate dehydrogenase (4-7). 

In a developing country with limited resources, we need a simple and readily available laboratory examination that can serve as a prognostic factor for hospitalized ESKD patients with COVID-19. 

The unique role of eosinophil in either COVID-19 or ESKD patients has been shown in several studies, but the results were inconsistent. Low eosinophil count, eosinopenia, was an indicator of diagnosis and related to disease severity and mortality of COVID-19 (8-11). A study showed that most deceased COVID-19 patients initially presented with eosinopenia remained eosinopenic compared with survivors (12). In contrast, a high eosinophil count, eosinophilia, was typical in ESKD patients undergoing HD. It may be due to their response to the HD circuit (13). Moreover, peripheral eosinophilia was associated with a higher risk of ESKD (14). The low level (<0.10 x10^3^/L) and high level (>0.55 x10^3^/L) of eosinophil were associated with mortality in HD patients. The characteristic of eosinophil count in ESKD patients corresponds reverse J-shape relationship (15). There is still a controversy regarding the implication of eosinophil in COVID-19, and there is limited data on the role of eosinophil in ESKD patients who contracted COVID-19. We aim to explore the risk factor of COVID-19 mortality in ESKD patients by comparing the survivors and nonsurvivors. We further investigate the value of eosinopenia as a prognostic marker of mortality in ESKD patients with COVID-19.

## Methods

**Study design and patients: **It is a retrospective study of COVID-19 in ESKD patients hospitalized after presenting to the Emergency Department. Data were revised from medical records of consecutive patients hospitalized in a tertiary referral hospital in West Nusa Tenggara, Indonesia, from May 2020 to October 2021. The inclusion criteria were: 1) newly diagnosed ESKD patients with an estimated glomerular filtration rate < 15 ml/minute (CKD-EPI equation) or patients on regular HD; 2) age > 18 years old; 3) a single positive RT-PCR test of SARS-CoV-2 infection from nasopharyngeal swab specimen; 4) data on complete blood count at presentation was available; 5) data in-hospital outcome was available. The exclusion criteria were patients discharged against medical advice before the outcome and missing data of RT-PCR or complete blood count at presentation. Demographic, history of contact, COVID-19 vaccination status, clinical data, laboratory results, and chest X-ray findings were collected on admission. The patients were categorized into eosinopenia and non-eosinopenia groups for the risk factor. The patients were divided into survival (S) and nonsurvival (NS) groups regarding the outcome. The study was approved by the Ethics Committee of West Nusa Tenggara Hospital (the code of ethics no. 070.1/37/KEP/2022), and the written informed consent was waived since this study is retrospective.

**Data analysis: **Comparative analysis was carried out between the death dan survival group. Continuous variable data were presented as mean standard deviation (SD) or median and inter-quartile range (IQR). Differences in quantitative parameters were assessed using the independent sample t-test or the Mann-Whitney U test. Categorical variable data were presented as n (%). Differences in qualitative parameters were assessed using the X^2^ and Fisher's exact test, as appropriate. Numerical independent variables that showed significant results in bivariate analysis were grouped into categorical variables before logistic regression analysis was performed. Logistic regression analysis was done to investigate the role of eosinopenia on the outcome after controlling other significant variables. The logistic regression analysis included important factors with p<0.05 in bivariate analysis. All data analyses were performed using SPSS statistical software package (Version 25.0; Chicago, IL), and p<0.05 was considered significant. 

## Results

One hundred twenty-two confirmed COVID-19 ESKD patients were admitted to our hospital during the 18-month period. Seven patients were excluded since five patients were discharged against medical advice, and two patients had data on RT-PCR missing. Finally, there were 115 patients included in the analyses. The average age was 50, 53% of patients were males, and 41% were newly diagnosed with ESKD. 

In HD patients, the median HD vintage was nine months, and the majority (63%) had an arteriovenous fistula. Only 14 patients had a contact history with positive COVID-19 patients or families.

 None of the patients had a history of COVID-19 vaccination. The underlying ESKD was diabetic nephropathy (32%), hypertensive nephrosclerosis (24%), and chronic glomerulonephritis (23%). The common comorbidities were hypertension (62%), diabetes mellitus (30%), and cardiovascular disease (15%). The frequent admission complications were pulmonary edema (28%), hyponatremia (24%), and hyperkalemia (17%). This study's mortality rate of COVID-19 in ESKD patients was 25.2%.

Clinical presentations between survivors and nonsurvivors were compared and analyzed (table 1). There was no significant difference between the survived and deceased patients regarding underlying ESKD, history of HD, comorbidity, and admission complications. The patients who died were significantly older than the surviving patients.

 Both the systolic and diastolic blood pressure of the death group were lower than the survival group. The nonsurvivor presented with a higher respiratory rate and lower peripheral oxygen saturation.

Laboratory markers of the deceased patients showed a higher neutrophil-to-lymphocyte ratio (NLR), a lower eosinophil count, and a higher C-reactive protein (CRP) level. 

Comparison between survivor and nonsurvivor was performed on categorized numerical variables. The proportion of high systolic blood pressure (SBP >140 mmHg) (S: 64.0%, NS: 62.1%; P=0.855) and elderly patients (Age > 60 years) (S: 15.1%, NS: 31.0%; P=0.059) were similar between the two groups. The proportion of low diastolic blood pressure (DBP <90 mmHg) (S: 57.0%, NS: 86.2%; P=0.004), tachypnea (respiratory rate >22 x/minutes) (S: 38.4%, NS: 65.5%; P=0.011), hypoxia (oxygen saturation <93%) (S: 19.8%, NS: 44.8%; P=0.008), high NLR (>6) (S: 43.0%, NS: 82.8%; p<0.001), eosinopenia (eosinophil count <0.01 x10^3^/uL) (S: 20.9%, NS: 65.5%; p<0.001), and high CRP (>20 mg/L) (S: 62.8%, NS: 82.8%; P=0.047) was significantly higher in the nonsurvivor than the survivor. 

**Table 1:** Comparison between the survival and nonsurvival of end-stage kidney disease patients with COVID-19

**Parameters**	**Survival** **(n = 86)**	**Nonsurvival (n = 29)**	**P-value**
**Demographics**			
Age, yr*	48 12	56 10	<0.001
**Men**	43 (50.0)	18 (62.1)	0.260
**Clinical presentations**			
**Fever**	18 (20.9)	11 (37.9)	0.068
**Cough**	32 (37.2)	11 (37.9)	0.945
**Dyspnea**	43 (50.0)	19 (65.5)	0.147
**Rhinorrhea**	4 (4.7)	2 (6.9)	0.641
**Abdominal pain**	8 (9.3)	7 (24.1)	0.056
**Myalgia**	9 (10.5)	2 (6.9)	0.728
**Fatigue/malaise**	51 (59.3)	13 (44.8)	0.175
**Nausea/vomiting**	25 (29.1)	7 (24.1)	0.608
**Other symptoms**	14 (16.3)	9 (31.0)	0.086
**Vital signs**			
Systolic blood pressure, mmHg*	155 34	138 25	0.004
**Diastolic blood pressure, mmHg**	87 (55-129)	78 (31-92)	0.001
**Pulse rate, beats per minute**	95 (63-185)	97 (56-121)	0.383
**Respiration rate, breaths per minute**	22 (18-36)	25 (20-50)	0.020
**Temperature, °C**	36.5 (36.0-38.8)	36.7 (36.0-38.7)	0.164
**Pulse oximetry, %**	97 (65-99)	92 (68-98)	0.001
Laboratory findings**			
**White blood cells, 10** ^3^ **/L**	9.2 (4.3-31.7)	9.1 (2.7-25.1)	0.317
Hemoglobin, g/dl*	8.7 1.9	9.3 2.7	0.199
**Thrombocyte, 10** ^3^ /L*	245 129	200 102	0.093
**Monocyte, 10** ^3^ **/L**	0.64 (0.11-2.12)	0.53 (0.13-1.15)	0.078
**Basophil, 10** ^3^ **/L**	0.03 (0.01-0.16)	0.03 (0.01-0.07)	0.119
**Eosinophil, 10** ^3^ **/L**	0.20 (0.01-1.36)	0.01 (0.00-0.09)	<0.001
**Neutrophil-to-lymphocyte ratio**	5.2 (1.5-55.4)	12.3 (2.2-49.3)	<0.001
**Estimated Glomerular Filtration Rate, ml/1,73m** ^2^ **/minute**	5.4 (1.5-14.5)	6.6 (2.0-14.8)	0.210
**Glucose, mg/dl**	129 (75-517)	124 (80-294)	0.206
**Sodium, mmol/L**	133 (100-142)	133 (120-144)	0.478
**Potassium, mmol/L**	4.6 (3.0-6.9)	4.4 (3.8-5.9)	0.877
C-reactive protein, mg/L, n=107***	26 (5-490)	120 (35-510)	<0.001
**Abnormal Chest X-ray findings**	73 (84.9)	26 (89.7)	0.758
**Management**			
**Antivirus**	81 (94.2)	26 (89.7)	0.414
**Oral anti-hypertension**	55 (64.0)	14 (48.3)	0.136
**Isolation Hemodialysis therapy**	81 (94.2)	17 (58.6)	<0.001

*Analyzed using independent t test; **Analyzed using Mann–Whitney U test; ***Indicates missing in 7% of patients

Standard treatments, including oxygen supplementation, antivirus, and antibiotics, were comparable between the survival and death groups. Antivirus was given to almost all patients consisting of 80% receiving oseltamivir, 10.5% receiving favipiravir, and 4.3% receiving remdesivir. None of the patients in this study received COVID-19-specific therapies such as tocilizumab, convalescent plasma, and intravenous immunoglobulin.

 Regarding the type of antibiotics, almost half of the patients were administered quinolones, 45.2% of patients received cephalosporins, and 5.2% of them were given a combination of cephalosporin and macrolides. There was no significant difference in HD prescription between the survivor and nonsurvivor. The median nucleic acid conversion time was 11 (5-60) days. The median time until discharge was 15 (8-46) days after admission in the survivors, and the median time to death in the nonsurvivors was four (1-36) days after admission.

The logistic regression analysis included several independent variables significantly correlated to death based on the univariate analysis (p < 0.05). Low diastolic blood pressure, tachypnea, hypoxia, high NLR, eosinopenia, high CRP, and isolation HD treatment were independently associated with mortality of COVID-19 in ESKD patients. However, on multivariate logistic regression analysis, eosinopenia and isolation HD therapy were risk factors that remained significant prognostic mortality factors (table 2).

**Table 2:** Univariable and multivariable analyses of odds ratio for mortality of COVID-19 in end-stage kidney disease patients

	**Univariable analysis**			**Multivariable analysis**		
	**OR**	**95% CI**	**P-value**	**OR**	**95% CI**	**P-value**
Low DBP	4.719	1.512-14.735	0.004	2.560	0.668-9.813	0.170
Tachypnea	3.052	1.265-7.360	0.011	3.153	0.844-11.789	0.088
Hypoxia	3.298	1.335-8.144	0.008	1.466	0.392-5.487	0.570
High NLR	6.357	2.216-18.235	<0.001	1.685	0.456-6.216	0.434
Eosinopenia	7.178	2.845-18.107	<0.001	4.180	1.264-13.826	0.019
High CRP	2.844	0.987-8.194	0.047	1.575	0.323-7.673	0.574
Isolation HD treatment	11.435	3.560-36.733	<0.001	14.659	2.955-72.726	0.001

DBP, diastolic blood pressure; NLR, neutrophil-to-lymphocyte ratio; CRP, C-reactive protein; OR, odds ratio; CI, confidence interval.

## Discussion

The mortality rate of COVID-19 in ESKD patients in Wuhan was higher than in the general population (1, 16). The mortality rate of COVID-19 in ESKD patients in this study was 25.2%. Several studies reported that the mortality rate of COVID-19 in the ESKD population differs from 3.6 to 31% (7, 17-20). The difference in mortality rate may be due to differences in inclusion criteria and sample size. The in-hospital length of stay for COVID-19 in ESKD patients ranges from 8-13 days, which is in line with the findings in this study (17-19, 21). 

Many studies report old age as a risk factor for COVID-19 death in EKSD but not this study (2, 6, 22, 23). The morbidity and mortality rate of COVID-19 in elderly patients was higher, presumably because the Angiotensin-Converting Enzyme-2 (ACE-2) receptors are less expressed in geriatric patients (21, 24). Over 85% of HD patients have one or more comorbid conditions (16, 25). Like this study, Valeri et al. reported that almost all patients had hypertension, 69% diabetes, and 46% coronary artery disease (19). The death group had more comorbidities and complications than the survival group, which differs from this study (19, 25). 

The main complication at admission in this study was pulmonary edema. It might result from patient factors such as inadequate HD, non-compliance to routine HD schedule, or fear of contracting COVID-19 in the hospital (17). Only 59% of patients in this study were on routine HD, whereas a study in the ESKD population in Saudi Arabia reported that most patients had regular HD (2). In contrast to this study, a study in Spain stated that HD vintage in the nonsurvivor group was longer than in the survivor group (4). A study in New York showed that the nonsurvivor presented with a higher respiratory rate and lower pulse oximetry than the survivor, similar to this study (17). Low oxygen saturation levels and low systolic and diastolic blood pressures were reported as independent risk factors for death from COVID-19 in HD patients (18, 23). A study in Belgium found that the nonsurvivor COVID-19 had a more significant proportion of patients with oxygen saturation <93% on presentation (21). Isolation HD therapy was a significant risk factor for death in ESKD patients with COVID-19. Immediate initiation of HD in ESKD patients with COVID-19 significantly improved outcomes (5, 19). Although HD was indicated in all hospitalized ESKD patients in this study, HD initiation was often delayed due to patient conditions, family concerns, and various reasons. Conservative treatment was continued while waiting for family approval. Finally, the patient's hemodynamics was unstable and did not meet the requirements for intermittent HD. Our hospital does not offer sustained low-efficiency HD and continuous renal replacement therapy. ESKD patients who contracted COVID-19 took longer to clear the virus; therefore, they needed extended isolation periods (26, 27). A study in Wuhan by Zhou et al. reported that viral shedding still occurred on the 37^th^ day of infection with a median of 20 days (24). A study by Dudreuilh et al. said that 41% of ESKD patients still showed positive PCR swabs on day 15, then negative at a median of 18 days (27). The median period of virus shedding in HD patients in the Wuhan study was 25 days, which aligns with our findings (23). 

Laboratory examination at admission can help predict the outcome of COVID-19 in ESKD patients. This study revealed that the death group had a lower lymphocyte count, higher NLR, and higher CRP levels than the survival group. A meta-analysis showed that the nonsurvivor group had higher leukocyte levels (28). Similarly, lymphopenia in the death group was reported in other studies (4, 5, 17, 29). Elevated CRP levels predicted COVID-19 mortality in HD patients (2, 4, 6, 18, 22). Little has been discussed about the role of eosinophils in patients with kidney disease. ESKD patients are generally accompanied by a condition of increased levels of eosinophils, which is commonly called eosinophilia. This eosinophil increase is also associated with inflammation, disease progression, and death in ESKD patients (14, 15). HD patients generally have an increased number of eosinophils because it is thought to be related to the body's reaction to the hemodialysis circuit. Persistent eosinophilia is characterized by the number of eosinophils >1 x10^9 ^/L) for three months. The proportion of persistent eosinophilia increased with the time patients had HD (13). 

Many studies reported eosinophil counts as a diagnostic tool for COVID-19 and a predictor of disease severity and poor prognosis in COVID-19 (9, 10, 12, 30, 31). Eosinophils benefit from controlling exacerbations of inflammation induced by neutrophils in COVID-19 patients. There was a negative correlation between the number of eosinophils and the number of neutrophils and NLR (9). Eosinophil levels correlate with organ disorders parameters. The lower the eosinophil, the higher the urea, creatinine, aspartate aminotransferase, lactate dehydrogenase, and creatinine kinase (32). Based on the severity, there was a significant difference in the mean number of eosinophils in peripheral blood in COVID-19 patients (31). 

This study revealed that the prognostic role of eosinophils in COVID-19 patients was identical to their role in ESKD patients contracting COVID-19. Eosinopenia had a negative effect, whereas eosinophilia had a protective effect on COVID-19. There were several functions of eosinophils in the COVID-19 process. Eosinophils could move to inflame loci during the resolution phase, produce anti-inflammatory and pro-resolving lipid mediators, counter-regulate neutrophil entry, stimulate neutrophil apoptosis, and upregulate phagocyte clearance through the lymphatic system (9).

This study reported the consistent role of eosinopenia in the mortality of COVID-19, particularly in ESKD patients. Eosinopenia was frequent and often severe in COVID-19 patients (31). 

Approximately 86% of COVID-19 patients who died came with laboratory results of eosinopenia and remained eosinopenia until the outcome occurred. This condition is known as persistent eosinopenia (12). A study in Italy reported that patients with absolute eosinopenia had a higher mortality rate and lower cure rate than patients without absolute eosinopenia. Of all the complete blood count parameters, only eosinopenia was an independent factor associated with mortality based on logistic regression analysis results (11). On the contrary, studies in India reported that 79.25% of COVID-19 patients with eosinopenia on admission to the hospital, and there was no significant difference in the median number of eosinophils between survivors and nonsurvivors. Likewise, there was no difference in the trend of eosinophils during treatment between the two groups (30). 

There were several limitations in this study. The small sample size was due to the limited number of cases from one single center. Detailed information on the kinetic of eosinophil count can not be analyzed since laboratory monitoring was performed at different points during hospitalization. Data regarding the exact reasons for the family and patient delaying HD initiation were unavailable.

In conclusion, eosinopenia was common in ESKD patients with COVID-19, particularly in the death group. Eosinopenia and HD therapy were significant risk factors for COVID-19 mortality in ESKD patients. Eosinopenia could represent reliable and quickly accessible prognostic indicators to help manage COVID-19 in ESKD patients. Early initiation of HD could improve the outcome of hospitalized ESKD patients with COVID-19. The kinetics of eosinophil counts during treatment and their relationship to clinical outcomes need to be explored in future studies.
